# Convergent functional change of frontoparietal network in obsessive-compulsive disorder: a voxel-based meta-analysis

**DOI:** 10.3389/fpsyt.2024.1401623

**Published:** 2024-07-08

**Authors:** Jianping Yu, Qianwen Xu, Lisha Ma, Yueqi Huang, Wenjing Zhu, Yan Liang, Yunzhan Wang, Wenxin Tang, Cheng Zhu, Xiaoying Jiang

**Affiliations:** ^1^ Affiliated Mental Health Center & Hangzhou Seventh People’s Hospital, Zhejiang University School of Medicine, Hangzhou, Zhejiang, China; ^2^ School of Psychology, Nanjing Normal University, Nanjing, China

**Keywords:** obsessive-compulsive disorder, magnetic resonance imaging, resting state, frontoparietal network, fronto-striatal circuit, meta-analysis

## Abstract

**Background:**

Obsessive-compulsive disorder (OCD) is a chronic psychiatric illness with complex clinical manifestations. Cognitive dysfunction may underlie OC symptoms. The frontoparietal network (FPN) is a key region involved in cognitive control. However, the findings of impaired FPN regions have been inconsistent. We employed meta-analysis to identify the fMRI-specific abnormalities of the FPN in OCD.

**Methods:**

PubMed, Web of Science, Scopus, and EBSCOhost were searched to screen resting-state functional magnetic resonance imaging (rs-fMRI) studies exploring dysfunction in the FPN of OCD patients using three indicators: the amplitude of low-frequency fluctuation/fractional amplitude of low-frequency fluctuation (ALFF/fALFF), regional homogeneity (ReHo) and functional connectivity (FC). We compared all patients with OCD and control group in a primary analysis, and divided the studies by medication in secondary meta-analyses with the activation likelihood estimation (ALE) algorithm.

**Results:**

A total of 31 eligible studies with 1359 OCD patients (756 men) and 1360 healthy controls (733 men) were included in the primary meta-analysis. We concluded specific changes in brain regions of FPN, mainly in the left dorsolateral prefrontal cortex (DLPFC, BA9), left inferior frontal gyrus (IFG, BA47), left superior temporal gyrus (STG, BA38), right posterior cingulate cortex (PCC, BA29), right inferior parietal lobule (IPL, BA40) and bilateral caudate. Additionally, altered connectivity within- and between-FPN were observed in the bilateral DLPFC, right cingulate gyrus and right thalamus. The secondary analyses showed improved convergence relative to the primary analysis.

**Conclusion:**

OCD patients showed dysfunction FPN, including impaired local important nodal brain regions and hypoconnectivity within the FPN (mainly in the bilateral DLPFC), during the resting state. Moreover, FPN appears to interact with the salience network (SN) and default mode network (DMN) through pivotal brain regions. Consistent with the hypothesis of fronto-striatal circuit dysfunction, especially in the dorsal cognitive circuit, these findings provide strong evidence for integrating two pathophysiological models of OCD.

## Introduction

Obsessive–compulsive disorder (OCD) is a common and destructive disorder with a lifetime prevalence of 1%-3% (1), characterized by intrusive thoughts (obsessions) and mental or physical ritualistic behaviors (compulsions). Cognitive rigidity is an important feature of OCD. Studies have shown that impaired cognitive flexibility is one of the core cognitive bases of OCD, which is closely associated with the dysfunction of the frontoparietal network (FPN) (2, 3). The FPN flexibly helps respond to stimuli or external tasks, and drives rapid, appropriate and purposeful coordinated behavior, which plays an important role in cognitive control (4, 5). Many studies have paid attention to functional alterations in the FPN in OCD using the resting-state functional magnetic resonance imaging (rs-fMRI), but consistent conclusions have not been reached. Therefore, we aimed to explore the fMRI-specific abnormalities of the FPN in OCD.

Rs-fMRI can detect deficits in disease-related neural activity in patients and is widely used in the study of the neural mechanism of OCD (6). The amplitude of low-frequency fluctuation (ALFF)/fractional amplitude of low-frequency fluctuation (fALFF), regional homogeneity (ReHo) and functional connectivity (FC) are currently the most commonly used methods to describe resting brain function. ALFF/fALFF and ReHo reflect the intensity and regional synchronization of spontaneous neural activity, respectively, providing information about local alterations in brain function (7). FC reflects the collaborative relationship between different brain regions, providing information about the global properties of intrinsically coupled brain networks (8). Thus, those methods obtaining functional information from multiple dimensions can investigate brain networks and understand the functional changes in OCD more comprehensively and deeply.

Previous rs-fMRI studies have reported abnormalities in intrinsic large-scale functional networks including the FPN, salience network (SN) and default mode network (DMN) in OCD patients (9). The FPN, as a key node in the triple network model (FPN, SN and DMN) linking with the classic fronto-striatal circuit, is regarded as the potential core of the pathophysiological mechanism of OCD (10). FPN comprises a wide-spread network including the dorsolateral prefrontal cortex (DLPFC), inferior parietal lobule (IPL) (11), inferior frontal gyrus (IFG) (12), superior temporal gyrus (STG) (13), middle temporal gyrus (MTG), supramarginal gyrus (14), posterior cingulate cortex (PCC, BA29) (15, 16), anterior cingulate cortex (ACC), frontal operculum (FO) and caudate (17). Several rs-fMRI studies have reported FPN hypoconnectivity (18, 19), hyperconnectivity (20–22) or even no significant (23) in patients with OCD compared with healthy controls (HCs). Previous studies on the FPN in OCD have shown inconsistent results. To date, no meta-analysis has focused on FPN of OCD. Certainly, two meta-analyses of seed-based resting-state functional connectivity in OCD have reported dysfunction in a portion of regions in the FPN. Gürsel reported FPN (mainly in the DLPFC) hypoconnectivity within-network and between-network with DMN (10). However, Liu only found dysconnectivity between the striatum and FPN (DLPFC and IPL) (24). A recent study, based on Gürsel’s meta-analysis results, conducted seed-to-voxel FC analyses and found no significant difference between OCD and HC in the FPN (14). Insufficient sample size and uncontrolled medication may have contributed to the observed differences. In addition, we suggested that the previous meta-analysis may be insufficient to summarize the FPN. It is necessitating further meta-analysis in FPN of OCD to quantify the evidence.

Thus, the main objective of the present study was to provide a contemporary, quantitative comparison of specific functional alterations of the FPN in OCD patients and HC by using the ALE algorithm. Further, we aimed to explore the changes within-network and between-networks in the FPN using three indicators (ALFF/fALFF, ReHo and FC). Based on previous knowledge, we hypothesized that (1) the ALFF/fALFF, ReHo and FC of the FPN would show special biomarkers, and (2) specific brain regions within the FPN would interact with other networks.

## Methods

### Literature search and study selection

We searched PubMed, Web of Science, Scopus, and EBSCOhost for studies in peer-reviewed journals through December 3rd, 2023. The search keywords were (obsessive–compulsive disorder or OCD) AND (functional magnetic resonance imaging or resting state) AND (functional connectivity or FC) AND (frontoparietal network) (2), (obsessive–compulsive disorder or OCD) AND (functional magnetic resonance imaging or resting state) AND (regional homogeneity or ReHo) (3), (obsessive–compulsive disorder or OCD) AND (functional magnetic resonance imaging or resting state) AND (amplitude of low-frequency fluctuations or ALFF or fractional amplitude of low-frequency fluctuations or fALFF).

The inclusion criteria were (1) reported comparisons of adult OCD patients and healthy controls (HC); (2) included an analysis of the FPN in the resting state;(3) employed whole brain analysis; (3) reported the peak coordinates of FPN regions in standard Montreal Neurological Institute (MNI) or Talairach space; and (4) published in English in peer-reviewed journals. Studies focused on pediatric populations, restricted to region of interest (ROI), did not report related peak coordinates were excluded. The reviews, meta-analyses and case reports were also excluded. If peak coordinates were not reported in the paper, we attempt to obtain them by contacting the corresponding author via email. And if there is overlap in the samples, the study with the larger sample size will be included in the meta-analysis.

Based on a preregistered protocol (PROSPERO CRD42023479734), this meta-analysis was conducted according to the PRISMA statement guidelines.

### Quality assessment and data extraction

The quality of each included study was assessed using a 12-point checklist (25) based on the reported demographic and clinical characteristics of the participants, as well as imaging methodology. Any study with a score >6.0 was included in the meta-analysis. More details are provided in the **Supplementary Material** (**Supplementary Table S1**).

For eligible study, clinical features (e.g. the sample size, mean age, sex composition, education level, mean age of onset, mean illness duration, mean total Yale-Brown Obsessive-Compulsive Scale (Y-BOCS) score, medication status and comorbidity) and neuroimaging characteristics (e.g. fMRI method, statistical threshold) were recorded. Then, the activity coordinates of the abnormal brain regions in FPN were used as inputs for GingerALE (26). Two researchers (JP. Y. and QW.X) independently conducted literature searches, study selection, data extraction and quality assessment. Any discrepancies were discussed with another author (C.Z.) to be mediated.

### Primary and secondary meta-analysis

The studies that met the inclusion criteria were all included in the primary meta-analysis, which explored characteristic changes in the FPN in patients with OCD. Furthermore, we were concerned about the impact of drugs on the results, prompting us to conduct secondary meta-analyses to evaluate potential effects in a drug-free group. Due to the limited number of included articles, we only included the ALFF and ReHO (OCD>HC) papers in the subgroup analysis.

### ALE meta-analysis

Voxel-based meta-analysis was performed using the GingerALE 3.0.2 software (http://www.brainmap.org), which is widely used in neuroimaging meta-analyses (27). Activation likelihood estimation (ALE) was used to assess the spatial convergence of the differences in ReHo, ALFF/fALFF and FC brain activity between OCD and HCs by analyzing the foci across studies using a random effects model. The extracted data was collated into six text files and entered into the ALE algorithm. The ALE algorithm considers the foci in text files as the spatial centers of the 3D Gaussian probability distribution and obtains the full-width half-maximum for the Gaussian distribution based on the sample size (27). Then, the ALE generated modeled activation (MA) maps and calculated ALE scores (28). The present study was conducted using a cluster-level inference corrected threshold of p < 0.05 (cluster-forming voxel p < 0.01, uncorrected) (29). The cluster-level inference corrected threshold sets the cluster minimum volume so that, for example, at a cluster-level inference threshold of 0.05, only 5% of the simulated data’s clusters exceed this size. Then, the DPABI software (http://fmri.org/dpabi) was used to visualize the ALE results. Finally, a jackknife sensitivity analysis was conducted by repeating the main analysis n times (where n equals the number of datasets included). In this analysis, one study at a time was excluded to assess if the results remained significant, thus evaluating the robustness of the results. However, due to the limited number of included studies, sensitivity analysis was only performed for the main meta-analysis.

## Results

### Studies included in meta-analysis

**Figure 1:**
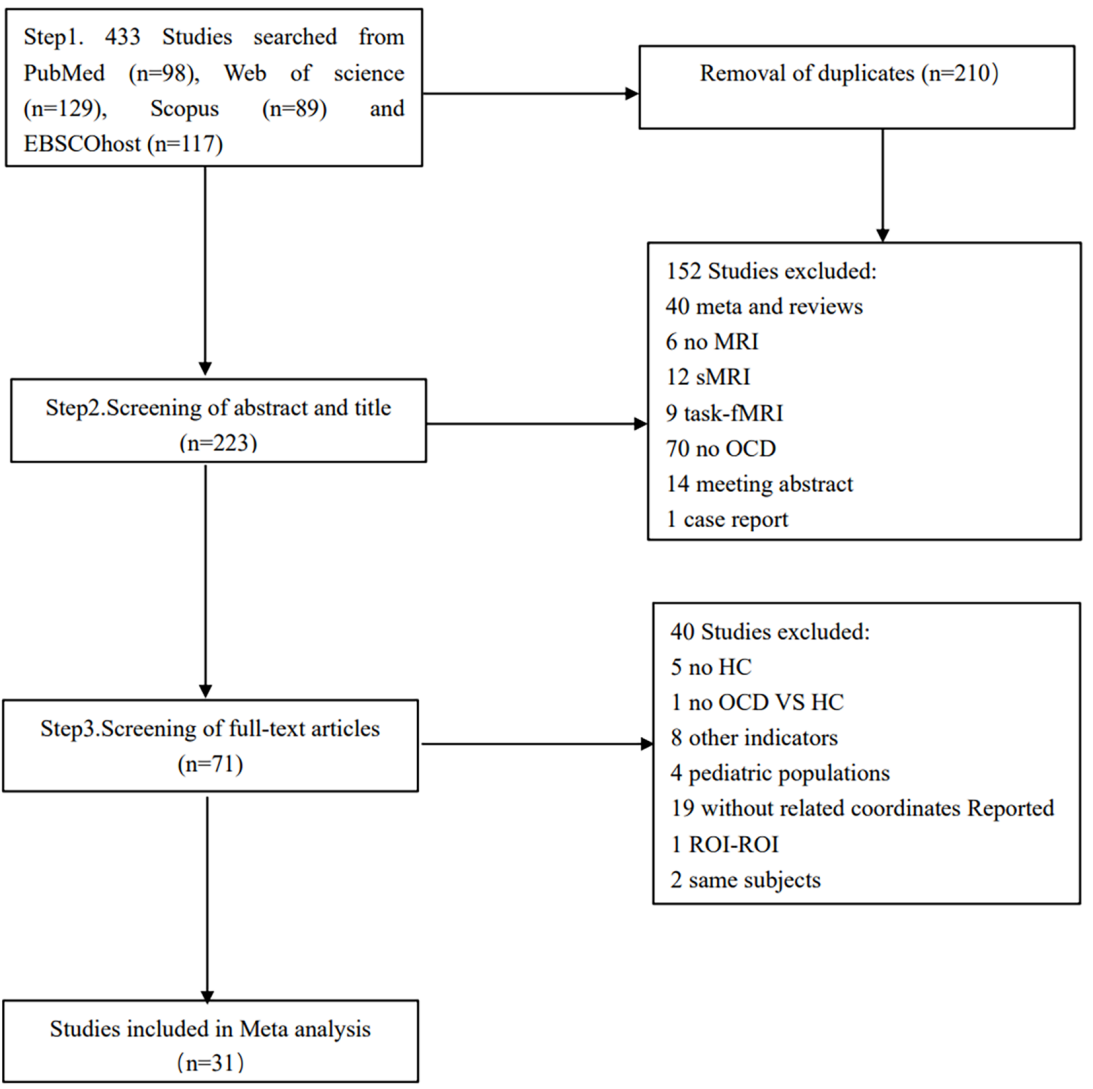
Flowchart of the procedures for the meta-analysis.

A total of 31 eligible studies comprising ALFF/fALFF 18 experiments, 1560 subjects and 59 foci; ReHo 14 experiments, 977 subjects and 63 foci; FC 12 experiments, 1149 subjects and 43 foci were included in the primary meta-analysis (**Table 1**). Six out of the 31 studies provided two experiments each. These studies typically contained two OCD subgroups, matched with only one HC group (40, 42, 44, 52–54). A total of 22 eligible studies comprising ALFF/fALFF 15 experiments, 1358 subjects and 50 foci; ReHo 9 experiments, 726 subjects and 29 foci were included in the secondary meta-analyses. The process of retrieval and selection is illustrated in the flowchart (**Figure 1**).

**Table 1:** Subject characteristics of the 31 studies included in meta-analysis.

Study	Methods	Sex	Age	Educ.	Age of	Illness	Total	Medication*	Comorbidity*
		(F/M)	(years)	(years)	onset*	Duration*	Y-BOCS*		
					(years)	(years)			
Yang et al., 2010 ^α^ (30)	ReHo	OCD (14,8)	31.18 ± 10.45	11.86 ± 3.56	12.14 ± 3.21	3.88 ± 4.08	32.27 ± 6.65	drug-free	None
		HC (14,8)	30.86 ± 9.07	12.14 ± 3.21					
Hou et al., 2012 ^α^ (31)	ALFF	OCD (11,10)	27.30 ± 9.90	11.90 ± 3.50	NA	5.34 ± 4.44	26.70 ± 6.10	drug-free	None
		HC (11,10)	26.00 ± 6.30	12.60 ± 3.90					
Cheng et al., 2013 ^α^ (32)	fALFF+	OCD (15,8)	31.00 ± 10.26	12.04 ± 3.78	27.04 ± 9.69	3.90 ± 4.48	31.61 ± 6.87	drug-free	None
	FC	HC (15,8)	31.65 ± 8.85	12.00 ± 3.38					
Ping et al., 2013 (33)	ReHo	OCD (4,16)	27.10 ± 8.00	14.2 ± 2.10	NA	7.34 ± 5.8	23.50 ± 5.80	6drug-free	None
		HC (4,16)	27.60 ± 8.20	14.0 ± 2.30				14under medication	
Yang et al., 2015 (34)	ReHo	OCD (10,12)	30.95 ± 8.69	14.24 ± 2.32	NA	8.22 ± 8.13	24.43 ± 5.99	10drug-free	None
		HC (10,12)	29.52 ± 7.96	15.34 ± 3.64				12under medication	
Chen et al., 2016 (35)	FC	OCD (6,24)	26.23 ± 5.69	NA	NA	5.54 ± 4.04	23.77 ± 6.85	10drug-free	None
		HC (7,23)	28.17 ± 7.65	NA				20under medication	
Niu et al., 2017 ^α^ (36)	ReHo	OCD (8,18)	24.19 ± 6.77	13.50 ± 2.83	NA	5.49 ± 6.19	22.92 ± 6.82	drug-free	None
		HC (13,12)	22.68 ± 4.96	15.04 ± 3.32					
Qiu et al., 2017 ^α^ (37)	fALFF	OCD (8,21)	26.60 ± 8.10	13.40 ± 2.90	NA	4.60 ± 4.70	22.50 ± 5.10	drug-free	None
		HC (8,21)	26.10 ± 7.90	14.40 ± 2.80					
Giménez et al., 2017 (38)	fALFF	OCD (29,36)	33.43 ± 8.20	13.09 ± 3.00	22.54 ± 7.90	11.19 ± 9.30	21.63 ± 6.20	7under medication	YES
		HC (20,30)	33.22 ± 10.40	12.62 ± 3.20				58under medication+CBT	
Zhao et al., 2017 ^α^ (39)	ALFF	OCD (13,18)	29.90 ± 8.00	13.60 ± 3.60	19.90 ± 7.70	9.80 ± 6.50	26.10 ± 5.30	drug-free	YES
		HC (11,14)	29.90 ± 8.70	14.00 ± 3.50					
Fan et al., 2017 (40)	ALFF	OCD-GI (10,9)	23.21 ± 6.35	13.53 ± 2.41	18.13 ± 3.86	NA	28.42 ± 6.72	17drug-free	None
		OCD-PI (6,12)	22.33 ± 6.21	12.69 ± 3.85	18.94 ± 4.14	NA	32.28 ± 5.71	20under medication	None
		HC (14,11)	23.88 ± 1.69	17.28 ± 1.81					
Li et al., 2019 ^α^ (41)	fALFF	OCD (26,19)	28.20 ± 8.67	11.27 ± 3.06	NA	3.16 ± 3.24	27.29 ± 6.55	drug-free	None
		HC (23,20)	28.30 ± 8.31	13.35 ± 2.75					
Xia et al., 2019 ^α^ (42)	ALFF	OCD-AH (17,12)	21.69 ± 5.31	13.17 ± 3.54	18.38 ± 4.42	4.14 ± 6.07	21.60 ± 4.90	drug-free	None
		OCD-NH (14,17)	23.77 ± 6.93	13.29 ± 3.22	19.29 ± 6.24	4.64 ± 4.46	19.23 ± 5.70		
		HC (17,13)	21.57 ± 2.46	15.50 ± 1.63					
Yang et al., 2019 ^α^ (18)	ReHo	OCD (9,6)	28.77 ± 6.84	12.46 ± 3.92	NA	7.15 ± 3.91	25.00 ± 6.29	drug-free	None
		HC (20,10)	28.23 ± 7.78	13.47 ± 2.99					
Hu et al., 2019 ^α^ (43)	ReHo	OCD (32,56)	29.16 ± 8.71	NA	21.84 ± 7.09	7.32 ± 5.58	21.47 ± 5.38	drug-free	None
		HC (32,56)	27.88 ± 10.58	NA					
Gao et al., 2019 ^α^ (17)	fALFF+	OCD (28,36)	29.00 ± 6.90	13.10 ± 1.10	NA	NA	23.50 ± 5.70	drug-free	None
	FC	HC (29,31)	28.50 ± 5.40	13.40 ± 1.20					
Yang et al., 2019 ^α^ (18)	fALFF+	OCD (23,45)	27.99 ± 8.19	13.83 ± 2.72	NA	6.40 ± 5.20	21.53 ± 5.38	drug-free	None
	FC	HC (23,45)	27.57 ± 8.57	13.25 ± 3.32					
Xia et al., 2020 ^α^ (44)	ReHo	OCD-AO (18,22)	22.48 ± 6.14	13.03 ± 2.84	18.05 ± 2.84	4.08 ± 4.58	21.63 ± 5.54	drug-free	None
		OCD-RO (21,21)	22.76 ± 6.14	12.61 ± 2.92	18.10 ± 5.70	4.33 ± 4.24	22.60 ± 5.45		
		HC (39,31)	20.93 ± 3.45	14.23 ± 2.62					
Gürsel et al., 2020 (45)	FC	OCD (33,16)	34.42 ± 12.07	NA	18.30 ± NA	NA	20.95 ± 6.10	18drug-free	YES
		HC (22,19)	35.07 ± 10.04	NA				31under medication	
Long et al., 2021 (46)	FC	OCD (12,19)	27.10 ± 9.50	13.70 ± 2.90	NA	6.00 ± 5.40	22.90 ± 5.20	drug-free	None
		HC (15,21)	24.60 ± 7.40	13.30 ± 2.80					
Yu et al., 2021 ^α^ (47)	ALFF+	OCD (6,21)	26.89 ± 8.15	13.26 ± 2.96	NA	3.00 ± NA	21.04 ± 5.93	drug-free	None
	ReHo	HC (22,38)	32.87 ± 10.78	14.02 ± 3.72					
Liu et al., 2021 ^α^ (48)	ALFF	OCD (30,43)	29.70 ± 8.51	NA	22.23 ± 7.21	7.47 ± 5.51	20.97 ± 5.26	drug-free	None
		HC (30,43)	28.19 ± 10.84	NA					
Zhang et al., 2021 (49)	FC	OCD (21,37)	27.20 ± 6.60	15.1 ± 2.80	19.60 ± 5.50	7.93 ± 5.58	21.46 ± 7.52	18drug-free	None
		HC (38,34)	24.40 ± 3.40	16.8 ± 2.10				40under medication	
Yan et al., 2022 ^α^ (50)	fALFF	OCD (14,20)	26.94 ± 8.79	13.68 ± 2.79	NA	4.73 ± 5.63	22.15 ± 5.13	drug-free	None
		HC (16,20)	24.19 ± 4.32	14.50 ± 1.52					
Han et al., 2022 ^α^ (51)	ALFF	OCD (47,52)	23.16 ± 9.34	11.95 ± 3.04	NA	4.01 ± 4.80	21.73 ± 6.91	drug-free	None
		HC (53,51)	23.14 ± 5.64	15.21 ± 3.17					
Yan et al., 2022 ^α^ (50)	ReHo	OCD (14,20)	26.94 ± 8.79	13.68 ± 2.79	NA	4.73 ± 5.63	22.15 ± 5.13	drug-free	None
		HC (16,20)	24.19 ± 4.32	14.50 ± 1.52					
Yu et al., 2022 (52)	ReHo+	Checker (11,19)	28.57 ± 8.01	15.67 ± 2.45	NA	NA	21.43 ± 5.88	30drug-free,	YES
	FC	Washer (8,7)	27.80 ± 7.70	14.87 ± 2.92	NA	NA	25.40 ± 5.12	15under medication	
		HC (23,22)	25.91 ± 3.85	15.89 ± 1.53					
Tomiyama et al., 2022 (20)	FC	OCD (29,18)	33.30 ± 11.87	NA	NA	NA	25.13 ± 5.73	drug-free	None
		HC (40,22)	32.61 ± 11.04	NA					
Ma et al., 2022 ^α^ (53)	ALFF+	mOCD (0,31)	28.90 ± 6.47	13.84 ± 2.12	4.95 ± 5.15	NA	26.70 ± 4.55	drug-free	None
	FC	fOCD (31,0)	28.76 ± 8.33	13.55 ± 3.16	6.13 ± 6.43		26.31 ± 5.83		
		HC (30,30)	30.78 ± 8.43	NA					
Yuan et al., 2023 ^α^ (54)	ReHo	OCDd (10,5)	15.60 ± 2.47	9.60 ± 2.47	NA	NA	22.53 ± 3.44	drug-free	None
		OCD (5,9)	15.64 ± 2.53	9.64 ± 2.53	NA	NA	19.79 ± 2.72		
		HC (6,11)	15.88 ± 1.83	9.88 ± 1.83					
Wu et al., 2023 (55)	FC	OCD (14,43)	28.12 ± 8.58	NA	NA	NA	27.42 ± 5.77	26drug-free	YES
		HC (22,51)	28.60 ± 8.25	NA			2.30 ± 3.39	31under medication	

Note: Values are mean (standard deviation); *OCD patients. α: subgroup of OCD patients with drug-free. OCD, obsessive-compulsive disorder; HC, healthy controls; Y-BOCS, Yale-Brown Obsessive-compulsive scale; NA, not available; F, female; M, male; ALFF, amplitude of low-frequency fluctuation; fALFF, fractional amplitude of low-frequency fluctuation, ReHo, regional homogeneity; FC, functional connectivity. OCD-GI, OCD with good insight; OCD-PI, OCD with poor insight; OCD-AH, OCD with anhedonia; OCD-NH, OCD with normal hedonic; OCD-AO, OCD with autogenous obsessions; OCD-RO, OCD with reactive obsessions; Checker, OCD with checking symptom; Washer, OCD with washing symptom; mOCD, male OCD; fOCD, female OCD; OCDd, OCD with depression.

### ALE meta-analysis results

We found specific changes in brain regions of FPN in OCD patients as compared to HC, mainly in the left dorsolateral prefrontal cortex (DLPFC, BA9), left inferior frontal gyrus (IFG, BA47), left superior temporal gyrus (STG, BA38), right posterior cingulate cortex (PCC, BA29), right inferior parietal lobule (IPL, BA40) and bilateral caudate. Additionally, altered connectivity within- and between-FPN were observed in the bilateral DLPFC, right cingulate gyrus and right thalamus.

#### Altered ALFF/fALFF in OCD

**Figure 2:**
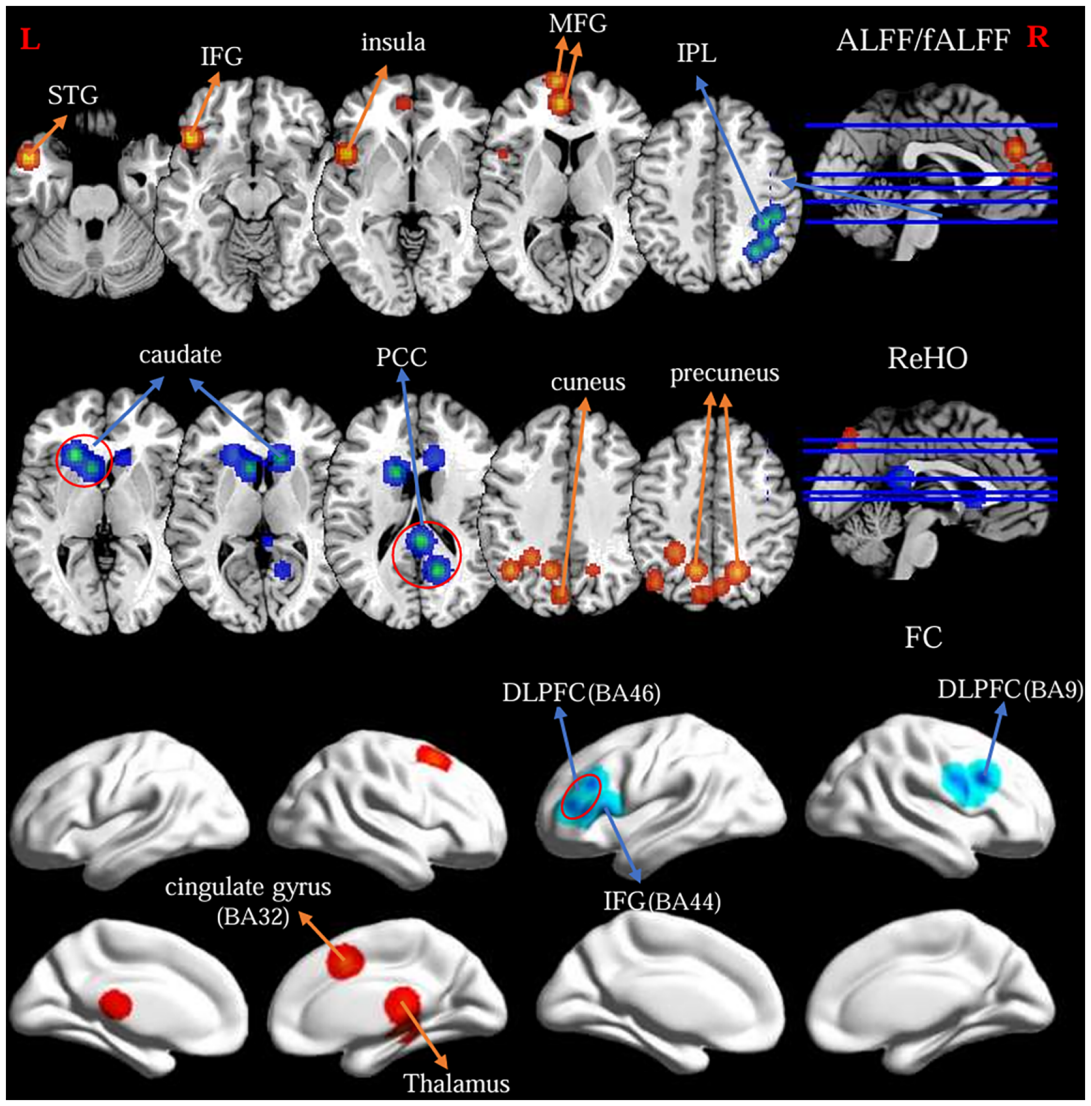
Findings from primary meta-analysis of the fMRI-specific differences of the FPN between OCD and HC. Warm/cool colors indicate regions showing activation/hyperconnectivity (or OCD > HC) and deactivation/hypoconnectivity (or OCD < HC), respectively.

The primary meta-analysis of the 15 studies revealed two clusters with a significant likelihood of higher activation (14 experiments comprising 26 foci and 1315 subjects) and a single cluster showing lower activation or “deactivation” (14 experiments comprising 33 foci and 1181 subjects). Compared with HC, OCD patients exhibited increased ALFF/fALFF in the left medial frontal gyrus (MFG, BA9 and 10), left inferior frontal gyrus (IFG, BA47), left superior temporal gyrus (STG, BA38) and left insula. Conversely, patients with OCD showed decreased ALFF/fALFF in the right inferior parietal lobule (IPL, BA40) and right precuneus (BA7) (**Table 2**; **Figure 2**).

**Table 2:** Regions with functional changes (ALFF, ReHo, and FC) from primary meta-analysis.

Cluster	voxels	ALE	MNI-coordinate			Brain region	Jacknife
	mm3		x	y	z		sensitivity
							analysis
ALFF/fALFF								
OCD>HC								
1	1380	0.009854	-6	46	24	Left MFG (BA9)	12/15
1		0.009706	-6	50	4	Left MFG (BA10)	12/15
2	1317	0.009904	-46	24	-14	Left IFG (BA47)	12/15
2		0.009903	-48	9	-27	Left STG (BA38)	12/15
2		0.009736	-48	12	-4	Left insula	12/15
HC>OCD								
1	1698	0.010428	36	-38	42	Right IPL (BA40)	11/15
1		0.010427	30	-60	45	Right precuneus (BA7)	11/15
ReHo								
OCD>HC								
1	2908	0.018498	-12	-60	40	Left precuneus (BA7)	11/11
1		0.012984	18	-62	46	Right precuneus (BA7)	9/11
1		0.010361	-6	-78	40	Left cuneus (BA19)	10/11
HC>OCD								
1	2668	0.009397	-14	12	18	Left caudate body	11/11
1		0.009285	-20	24	0	Left caudate head	11/11
1		0.009066	13	22	9	Right caudate body	9/11
2	1441	0.009066	3	-39	16	Right PCC (BA29)	11/11
2		0.008955	15	-60	15	Right PCC (BA30)	11/11
FC								
OCD>HC								
1	1408	0.010084	12	12	42	Right cingulate gyrus	10/11
						(BA32)	
2	1320	0.010539	18	-26	-2	Right Thalamus	9/11
HC>OCD								
1	1755	0.010359	-46	28	26	Left DLPFC (BA46)	11/11
1		0.01026	-48	18	10	Left IFG (BA44)	11/11
2	1376	0.010447	56	10	22	Right DLPFC (BA9)	9/11
2		0.010315	40	18	30	Right precentral gyrus	9/11

MFG, medial frontal gurus; IFG, inferior frontal gyrus; STG, superior temporal gyrus; IPL, inferior parietal lobule; PCC, posterior cingulate cortex; DLPFC, dorsolateral prefrontal cortex.

The secondary meta-analyses of the 13 studies identified two clusters with a significant likelihood of higher activation (12 experiments comprising 23 foci and 1156 subjects) and two clusters showing lower activation or “deactivation” (10 experiments comprising 27 foci and 937 subjects). In addition to replicating the primary analysis results, the secondary analysis also revealed that OCD showed decreased ALFF/fALFF in the left insula(BA13) and left STG (BA41) compared to HC (**Table 3**).

**Table 3:** Regions with functional changes (ALFF and ReHo) from secondary meta-analyses.

Cluster	voxels	ALE	MNI-coordinate			Brain region
	mm3		x	y	z	
ALFF/fALFF							
drug-free OCD>HC							
1	1612	0.009854	-6	46	24	Left MFG (BA9)
1		0.009706	-6	50	4	Left MFG (BA10)
2	1501	0.009904	-46	24	-14	Left IFG (BA47)
2		0.009903	-48	9	-27	Left STG (BA38)
2		0.009736	-48	12	-4	Left insula
HC>drug-free OCD							
1	1991	0.010428	36	-38	42	Right IPL (BA40)
1		0.010427	30	-60	45	Right precuneus (BA7)
2	1373	0.015433	-38	-28	16	Left insula (BA13)
2		0.010059	-41	-30	6	Left STG (BA41)
ReHo							
drug-free OCD>HC							
NA						

NA, not available.

MFG, medial frontal gurus; IFG, inferior frontal gyrus; STG, superior temporal gyrus; IPL, inferior parietal lobule.

#### Altered ReHO in OCD

The primary meta-analysis of the 11 studies revealed a single cluster showing a significant likelihood of higher activation (13 experiments comprising 47 foci and 945 subjects) and two clusters indicating lower activation or “deactivation” (7 experiments comprising 16 foci and 311 subjects). OCD patients exhibited increased ReHo in the bilateral precuneus (BA7) and left cuneus (B19). Besides, patients with OCD showed decreased ReHo in bilateral caudate body, left caudate head and right posterior cingulate cortex (BA29 and 30) (**Table 2**; **Figure 2**).

The secondary meta-analyses of the 8 studies comprising 9 experiments, 726 subjects and 29 foci did not identified clusters (**Table 3**).

#### Altered FC in OCD

ALE meta-analysis of the 11 studies revealed three clusters with a significant likelihood of strengthened connectivity (8 experiments comprising 18 foci and 725 subjects) and four clusters of weakened connectivity (7 experiments comprising 25 foci and 654 subjects). The results showed increased FC in the right cingulate gyrus (BA32), right thalamus. Differently, patients with OCD showed decreased FC of the FPN in the bilateral dorsolateral prefrontal cortex (DLPFC, BA9 and 46), left inferior frontal gyrus (IFG, BA44) and right precentral gyrus (BA9) (**Table 2**; **Figure 2**).

#### Jackknife sensitivity analysis

The jackknife sensitivity analysis showed that the decreased FC in the left DLPFC/IFG, the increased ReHo in the left precuneus and the decreased ReHo in the left caudate were the most robust and replicable data. Moreover, the increased FC in the right cingulate gyrus remained highly replicable (**Table 2**). More details are provided in the **Supplementary Material** (**Supplementary Table S2**).

## Discussion

Our meta-analysis was the first to assess the functional integrity of the FPN in OCD using fMRI across various dimensions (ALFF/fALFF, ReHo and FC). We found that hypoconnectivity in the bilateral dorsolateral prefrontal cortex (DLPFC, BA9 and 46) is the most central feature of the FPN. Moreover, the dysfunction between FPN, fronto-striatal circuit, DMN and SN in OCD was reported. Further, we discussed the main findings.

### Dorsal striatum

In meta-analysis, the bilateral caudate body and left caudate head showed decreased ReHo in OCD compared to HC. This finding supports the important role of dorsal striatal dysfunction in the FPN of OCD. The caudate nucleus projects to the thalamus and is regulated by DLPFC, and together they form the dorsal cognitive circuit involved in executive functions and top-down control of emotional and motor processes (56). Abnormal activity in the caudate nucleus has been consistently reported in previous studies of OCD (57). It is associated with cognitive functions, such as response inhibition and flexibility (58), and emotion regulation (59). A recent rs-fMRI meta-analysis demonstrated lower left caudate activation in OCD vs. HC (60). This finding was consistent with our study, indicating reduced flexibility of response in OCD patients. Furthermore, hypoactivation of the right caudate body was observed in OCD during executive functioning tasks (61). Several others meta-analysis have also reported that hypoactivation of the bilateral caudate in inhibitory control (62) and the left caudate body in symptom provocation that elicits intense negative emotions (63). Thus, caudate abnormality appears to be a specific feature of OCD. We suggested that the decreased ReHo in the bilateral caudate body and left caudate head indicates poor coordination of local neural activity, which may be related to dysregulated emotion and behavior in OCD and plays a significant role in the pathophysiology of OCD.

### Thalamus

The increased FC in the right thalamus was reported in OCD patients relative to HC. The thalamus, as a relay station, plays a crucial role in fronto-striatal circuit of OCD. It integrates incoming sensory information from the basal ganglia, especially in the caudate nucleus, with higher cortical (DLPFC) functions, and participates in cognitive and motor functions (64). Inefficient thalamic gating, caused by the dysfunction of the caudate nucleus, leads to hyperactivation of the DLPFC (corresponding to intrusive thoughts) and ACC (associated with non-specific anxiety) (65). Previous studies have reported increased FC in the thalamus at rest (66, 67). The increased FC of the thalamus is often interpreted as a compensatory brain activity that activates connected brain regions and enhances the ability of information integration (68). Recent studies have also shown increased FC between the regions of the FPN (DLPFC) and the thalamus in OCD and their first-degree relatives, in line with the findings in our meta-analysis. It has been proposed that this may be a candidate endophenotype markers of OCD (20).

### Dorsolateral prefrontal cortex (DLPFC)

Our meta-analysis revealed a decreased FC of the FPN in the bilateral DLPFC (BA9 and 46) in OCD. The DLPFC is involved in executive functions and emotion regulation (69). It is a key node in the FPN involved in cognitive control and the fronto-striatal loop in habitual behavior (70, 71). The decreased FC in the bilateral DLPFC may indicate a top-down disruption between these networks and underlie the pathophysiology and clinical symptoms of OCD (72). Likewise, increased ALFF/fALFF in BA9 was also reported in OCD during rest in our results. This may be related to caudate hypofunction and thalamic gating dysfunctions, as mentioned previously. In addition, hyperactivation of the left BA9 may be observed as a compensatory activation that compensates for the dysregulation by recruiting cortical areas. The larger range of the frontal cortex, including the left MFG (BA9 and 10) and the left IFG (BA47), showed higher activation in OCD patients relative to HC in our study. OCD struggle to control compulsive thoughts, leading to heightened vigilance. This can overwhelm the executive system, deplete cognitive resources, worse cognitive impairment and emotional dysregulation, and trigger compulsive behaviors (64). This result is consistent with the finding that electrical and magnetic stimulation of the left DLPFC appeared to have clinical efficacy for treatment-resistant OCD (73).

### Cingulate cortex

The cingulate cortex, as part of the limbic system, is usually divided into the anterior region (ACC) and the posterior region (PCC), which have different functions and are implicated in the pathophysiology of OCD (74). The ACC is involved in executive control and PCC in evaluative functions and human awareness. Specifically, the ACC monitors self-generated threats by scanning the internal mental environment, whereas the PCC scans the external environment to monitor for environmental threats (75–77). Previous fMRI studies have reported ACC hyperactivation (31, 78) and PCC hypoactivation (49, 79) in OCD at rest, consistent with the observation of decreased ReHo in the right PCC (BA29 and 30) in our meta-analysis. Hypoactivation of the right PCC may be associated with diminished perception and attention to external events, which might explain the symptoms of intrusive thoughts and repetitive behavior in OCD (50). However, the higher ACC activation was not observed in our results, and we speculate that this may be related to the fact that some OCD patients are taking medication. As previously reported, treatment may reduce ACC spontaneous activity (80, 81).

However, ALE revealed increased FC in the right cingulate gyrus (BA32) in OCD than in HC. Previous studies have shown that the fronto-cingulate system plays a key role in error detection and control. Increased FC between the ACC and DLPFC is thought to be related to abnormal error processing in OCD patients (82). Other studies have shown that increased FC in the ACC was correlated with symptom severity in OCD patients (32). Hence, the current findings emphasize the important role of the ACC in cognitive control and further support the role of neural activity in the right cingulate cortex in the core deficits of OCD.

### Interactive neural network

Based on ALE results, we found characteristic alterations in the FPN of OCD, as well as underlying brain regions that interact with other networks, such as the DMN, SN and fronto-striatal loop. The triple network model and classical fronto-striatal circuit may underlie the pathophysiology of OCD (83, 84).Furthmore, the mate-analysis emphasizes the importance of the FPN in OCD pathology.

The dysfunction of the bilateral DLPFC (BA9 and 46), left IFG (BA47), right PCC (BA29), left STG, right IPL and bilateral caudate was reported in the FPN of OCD at rest. The FPN is a flexible cognitive control center that typically regulates and adjusts behavior in a goal-directed manner during both resting and task states (85). The IFG is the core system for goal-directed tasks, involved in response inhibition (86). The STG is associated with social cognition, particularly involving interpretation/speculation about the intentions or goal-directed behaviors of others (42, 87). The IPL is associated with attentional set shifting and response inhibition (88). Previous studies have suggested that hypoactivation of the IPL perhaps reflect impaired attention to stop signals in OCD, leading to compulsive behavior (48). These important nodes of the FPN exhibited varying degrees of dysfunction; specifically, reduced FC in the DLPFC, the core brain region of the FPN, may suggest indicate ineffective connections within the FPN. The abnormal connectivity pattern of the FPN is related to dysfunction of cognitive control (89). Moreover, a rs-fMRI study on OCD reported that the connectivity within-FPN is negatively associated with disease severity (22). It further emphasizes the important role this network plays in pathophysiology and clinical manifestations of OCD.

ALE showed greater FC in the right cingulate gyrus (BA32) in OCD in results of FC. The ACC is core region of the SN (45). Thus, this finding may indicate a stronger functional connectivity between the FPN and SN. Together, they form a “task-positive” system that supports cognitive control (71). We speculate that this may indicate inefficiency of the cognitive control networks in OCD patients due to the hypoconnectivity within FPN. Increased connectivity is considered a complementary mechanism (90) that enhances cognitive ability and maintains normal function. Generally, intrusive thoughts are more likely to occur when the mind is not engaged in cognitively demanding tasks, such as during rest (91, 92). Hence, OCD patients may experience more cognitive and emotional challenges during relaxation time, needing to suppress OC symptoms and anxious emotions in the resting state. The atypical recruitment of the “task-positive” system may help OCD patients control these thoughts and related avoidance behaviors (93). Besides, we also found higher right insula activation during the resting state in OCD from meta results. The insula is another core region of the SN, which is involved in detecting salient stimuli (94). The hyperactive insula may be linked to a stronger ability to perceive error-related signals, which can help patients with OCD to avoid making more mistakes (3).

The DMN is a significant large-scale intrinsic network involved in self-referential thoughts, mind-wandering and internal processes (95). The alterations of DMN function may be thought to underlie the intrusive thoughts and anxiety experienced in OCD patients (93). As the task-negative network, the DMN interacts reciprocally with the task-positive system (96). In our results, we did not directly find abnormal FC between FPN and DMN. However, local functional abnormalities in DMN core nodes (97), including MFG, PCC and precuneus, associated with the FPN were observed. There are also overlapping areas of the brain between the FPN (MFC, BA 9; PCC, BA29) and the DMN (MFC, BA10; PCC, BA30) in structure. We suggest that the FPN may directly influence DMN activity through these overlapping brain regions. Previous fMRI studies have confirmed that real-time regulation of the PCC can lead to change in activity in other brain regions and functional alterations of the DMN (98). Further, the FC between the FPN and DMN for OCD remains to be investigated.

The hypoconnectivity within the FPN, the better connectivity between FPN and SN, and the potential interaction between FPN and DMN were observed during the resting state in our study. Treatment may alter the connectivity within- and between-FPN in OCD. Some studies have reported that OCD patients showed significantly increased connectivity within the FPN after undergoing exposure and response prevention (ERP) therapy (71) and pharmacological (99) treatment. The greater connectivity within the FPN can enhance cognitive control functions (100). Some studies focusing on patients with attention deficit hyperactivity disorder (ADHD) found increased connectivity between the FPN and SN (101). This change in connectivity plays an important role in helping patients implements cognitive control. ADHD patients can be treated with working memory training to modulate widespread FPN and SN areas (102). However, the functional changes between FPN and SN after treatment in OCD need to be further studied. In addition, the decreased connectivity between FPN and DMN was observed in OCD following ERP treatment (71). This may be related to the fact that patients must repeatedly engage in cognitive control processes to resist compulsive impulses during treatment. Other studies have shown that the connectivity between DMN and FPN significantly predicted response to ERP (103).

**Figure 3:**
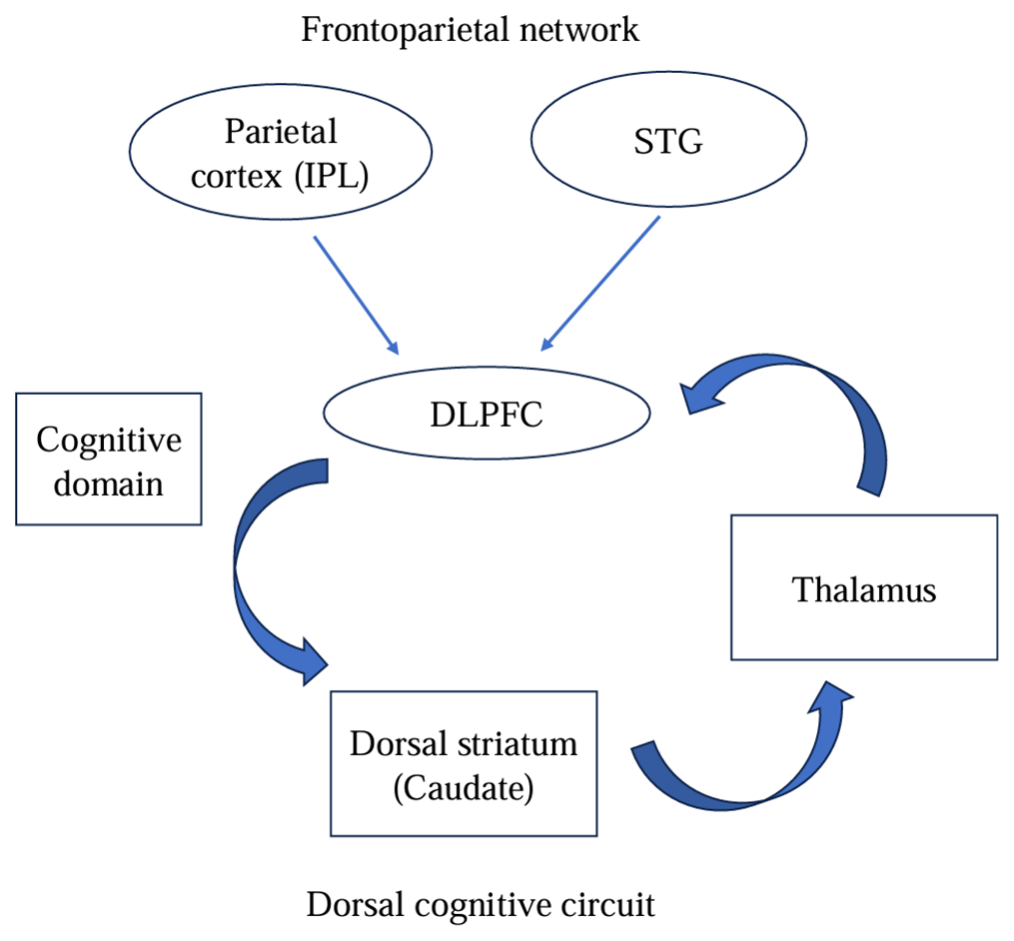
The cognitive domain: integrated model of pathophysiology in OCD.

In summary, the atypical recruitment of the FPN appears to couple with abnormal activity in the SN and DMN, resulting in impaired cognitive performance in OCD patients. Notably, the functional specific alterations of the FPN findings match the structures of the fronto-striatal circuit, especially in the dorsal cognitive circuit (DLPFC-caudate-thalamus) (56). The imbalance of functional activity within these two systems (**Figure 3**), the triple network model and the dorsal cognitive circuit, may reflect the essence of the pathophysiological of OCD and underlie the OC symptoms.

### Limitations

This study has several limitations. First, the number of studies was insufficient for symptom subtype analysis. Second, although we conducted subgroup analyses of drug-free OCD patients, the number of studies was limited, and it was still not possible to accurately assess the potential impact of the drug on the likelihood of activation. Future studies should increase the sample size, distinguish the symptom subtypes, and control for the influence of medication and comorbidity, which may facilitate a more thorough exploration of pathogenesis of OCD. Finally, we were unable to perform correlation analysis and confidence interval analysis due to the limitation of the ALE method.

## Conclusions

We conducted a meta-analysis to examine specific functional alterations of FPN in OCD patients compared to HC. The study observed the functional impairment and potential compensation mechanisms of the FPN. We concluded that OCD patients showed local brain functional changes, including higher activation in the left DLPFC (BA9), left IFG and left STG, as well as lower activation in the right IPL, right PCC (BA29) and bilateral caudate. Additionally, there was hypoconnectivity within the FPN, particularly in the bilateral DLPFC. And FPN appears to couple with the SN and DMN through pivotal brain regions, including right cingulate gyrus, left MFC, and right PCC. Moreover, these impaired brain areas overlap with the classic fronto-striatal circuit, especially in the dorsal cognitive circuit. Thus, two pathophysiological models of OCD could be integrated into a common framework to explain core OC symptoms.

## Author contributions

JY: Conceptualization, Data curation, Formal analysis, Methodology, Software, Visualization, Writing – original draft. QX: Data curation, Validation, Writing – review & editing. LM: Formal analysis, Writing – review & editing. YH: Formal analysis, Writing – review & editing. WZ: Supervision, Writing – review & editing. YL: Supervision, Writing – review & editing. YW: Formal analysis, Writing – review & editing. WT: Conceptualization, Writing – review & editing. CZ: Data curation, Funding acquisition, Supervision, Writing – review & editing. XJ: Conceptualization, Supervision, Writing – review & editing.
