# Supplementary material for: Convergent functional change of frontoparietal network in obsessive-compulsive disorder: a voxel-based meta-analysis
Source: Front Psychiatry. 2024 Jul 8;15:1401623. doi: 10.3389/fpsyt.2024.1401623 (PMC11260709; doi:10.3389/fpsyt.2024.1401623)
Supplement: Supplementary file 1 [file Table_1.docx]

Table S1. The checklist of imaging methodology quality assessment for all the articles included in meta-analysis.

| **Study** | **Category 1: Subjects** | | | | **Category 2: Methods for image acquisition and analysis** | | | | | | **Category 3: Results and conclusions** | |  |
| --- | --- | --- | --- | --- | --- | --- | --- | --- | --- | --- | --- | --- | --- |
|  | Patients were evaluated prospectively, specific diagnostic criteria were applied, and demographic data were reported | Healthy comparison participants were evaluated prospectively; psychiatric and medical illnesses were excluded | Important variables (e.g., age, gender, drug status, illness duration, and symptom severity) were checked either via stratification or statistics | Sample size per group: ≥ 20, scores 1; ≥ 10, scores 0.5 | All neuroanatomic measurements were made blind to group assignment and to subjects’ identity | Measures for brain structures were reported | Magnet strength: 3T, scores 1; 1.5T, scores 0.5 | The imaging technique used was clearly described so that it could be reproduced | Whole brain analysis was automated without a previously defined region | Spatial coordinates were reported in a standard space (e.g., Talairach or MNI coordinates) | Statistical results were corrected for multiple comparison scores 1, uncorrected scores 0.5 | Conclusions were consistent with the results obtained, and the limitations were discussed | total |
| Yang et al. 2010(1) | 1 | 1 | 1 | 1 | 1 | 1 | 0.5 | 1 | 1 | 1 | 1 | 1 | 11.5 |
| Hou et al. 2012(2) | 1 | 1 | 1 | 1 | 1 | 1 | 1 | 1 | 1 | 1 | 1 | 1 | 12 |
| Cheng et al. 2013(3) | 1 | 1 | 1 | 1 | 1 | 1 | 0.5 | 1 | 1 | 1 | 1 | 1 | 11.5 |
| Ping et al. 2013(4) | 1 | 1 | 1 | 1 | 1 | 1 | 1 | 1 | 1 | 1 | 1 | 1 | 12 |
| Yang et al. 2015(5) | 1 | 1 | 1 | 1 | 1 | 1 | 1 | 1 | 1 | 1 | 1 | 1 | 12 |
| Chen et al. 2016(6) | 1 | 1 | 1 | 1 | 1 | 1 | 1 | 1 | 1 | 1 | 1 | 1 | 12 |
| Niu et al. 2017(7) | 1 | 1 | 1 | 1 | 1 | 1 | 1 | 1 | 1 | 1 | 1 | 1 | 12 |
| Qiu et al. 2017(8) | 1 | 1 | 1 | 1 | 1 | 1 | 1 | 1 | 1 | 1 | 1 | 1 | 12 |
| Giménez et al. 2017(9) | 1 | 1 | 1 | 1 | 1 | 1 | 0.5 | 1 | 1 | 1 | 1 | 1 | 11.5 |
| Zhao et al. 2017(10) | 1 | 1 | 1 | 1 | 1 | 1 | 1 | 1 | 1 | 1 | 1 | 1 | 12 |
| Fan et al. 2017(11) | 1 | 1 | 0.5 | 0.5 | 1 | 1 | 1 | 1 | 1 | 1 | 1 | 1 | 11 |
| Li et al. 2019(12) | 1 | 1 | 1 | 1 | 1 | 1 | 1 | 1 | 1 | 1 | 0.5 | 1 | 11.5 |
| Xia et al. 2019(13) | 1 | 1 | 1 | 0.5 | 1 | 1 | 1 | 1 | 1 | 1 | 1 | 1 | 11.5 |
| Yang et al. 2019(14) | 1 | 1 | 1 | 0.5 | 1 | 1 | 1 | 1 | 1 | 1 | 1 | 1 | 11.5 |
| Hu et al. 2019(15) | 1 | 1 | 1 | 1 | 1 | 1 | 1 | 1 | 1 | 1 | 1 | 1 | 12 |
| Gao et al. 2019(16) | 1 | 1 | 0.5 | 1 | 1 | 1 | 1 | 1 | 1 | 1 | 1 | 1 | 11.5 |
| Yang et al. 2019(17) | 1 | 1 | 1 | 1 | 1 | 1 | 1 | 1 | 1 | 1 | 1 | 1 | 12 |
| Xia et al. 2020(18) | 1 | 1 | 1 | 1 | 1 | 1 | 1 | 1 | 1 | 1 | 1 | 1 | 12 |
| Gürsel et al. 2020(19) | 1 | 1 | 0.5 | 1 | 1 | 1 | 1 | 1 | 1 | 1 | 1 | 1 | 11.5 |
| Long et al. 2021(20) | 1 | 1 | 1 | 1 | 1 | 1 | 1 | 1 | 1 | 1 | 1 | 1 | 12 |
| Yu et al. 2021(21) | 1 | 1 | 0.5 | 1 | 1 | 1 | 1 | 1 | 1 | 1 | 1 | 1 | 11.5 |
| Liu et al. 2021(22) | 1 | 1 | 1 | 1 | 1 | 1 | 1 | 1 | 1 | 1 | 1 | 1 | 12 |
| Zhang et al. 2021(23) | 1 | 1 | 1 | 1 | 1 | 1 | 1 | 1 | 1 | 1 | 1 | 1 | 12 |
| Yan et al. 2022a(24) | 1 | 1 | 1 | 1 | 1 | 1 | 1 | 1 | 1 | 1 | 1 | 1 | 12 |
| Han et al. 2022(25) | 1 | 1 | 1 | 1 | 1 | 1 | 1 | 1 | 1 | 1 | 1 | 1 | 12 |
| Yan et al. 2022b(26) | 1 | 1 | 1 | 1 | 1 | 1 | 1 | 1 | 1 | 1 | 1 | 1 | 12 |
| Yu et al. 2022(27) | 1 | 1 | 0.5 | 0.5 | 1 | 1 | 1 | 1 | 1 | 1 | 1 | 1 | 11 |
| Tomiyama et al. 2022(28) | 1 | 1 | 0.5 | 1 | 1 | 1 | 1 | 1 | 1 | 1 | 1 | 1 | 11.5 |
| Ma et al. 2022(29) | 1 | 1 | 0.5 | 1 | 1 | 1 | 1 | 1 | 1 | 1 | 1 | 1 | 11.5 |
| Yuan et al. 2023(30) | 1 | 1 | 0.5 | 0.5 | 1 | 1 | 1 | 1 | 1 | 1 | 1 | 1 | 11 |
| Wu et al. 2023(31) | 1 | 1 | 0.5 | 1 | 1 | 1 | 1 | 1 | 1 | 1 | 1 | 1 | 11.5 |

1. Yang T, Cheng Y, Li H, Jiang H, Luo C, Shan B, et al. Abnormal Regional Homogeneity of Drug-Naïve Obsessive-Compulsive Patients. *Neuroreport* (2010) 21(11):786-90. Epub 2010/06/24. doi: 10.1097/WNR.0b013e32833cadf0.

2. Hou J, Wu W, Lin Y, Wang J, Zhou D, Guo J, et al. Localization of Cerebral Functional Deficits in Patients with Obsessive-Compulsive Disorder: A Resting-State Fmri Study. *Journal of affective disorders* (2012) 138(3):313-21. Epub 2012/02/15. doi: 10.1016/j.jad.2012.01.022.

3. Cheng Y, Xu J, Nie B, Luo C, Yang T, Li H, et al. Abnormal Resting-State Activities and Functional Connectivities of the Anterior and the Posterior Cortexes in Medication-Naïve Patients with Obsessive-Compulsive Disorder. *PloS one* (2013) 8(6):e67478. Epub 2013/07/11. doi: 10.1371/journal.pone.0067478.

4. Ping L, Su-Fang L, Hai-Ying H, Zhang-Ye D, Jia L, Zhi-Hua G, et al. Abnormal Spontaneous Neural Activity in Obsessive-Compulsive Disorder: A Resting-State Functional Magnetic Resonance Imaging Study. *PloS one* (2013) 8(6):e67262. Epub 2013/07/05. doi: 10.1371/journal.pone.0067262.

5. Yang XY, Sun J, Luo J, Zhong ZX, Li P, Yao SM, et al. Regional Homogeneity of Spontaneous Brain Activity in Adult Patients with Obsessive-Compulsive Disorder before and after Cognitive Behavioural Therapy. *Journal of affective disorders* (2015) 188:243-51. Epub 2015/09/18. doi: 10.1016/j.jad.2015.07.048.

6. Chen Y, Meng X, Hu Q, Cui H, Ding Y, Kang L, et al. Altered Resting-State Functional Organization within the Central Executive Network in Obsessive-Compulsive Disorder. *Psychiatry and clinical neurosciences* (2016) 70(10):448-56. Epub 2016/07/06. doi: 10.1111/pcn.12419.

7. Niu Q, Yang L, Song X, Chu C, Liu H, Zhang L, et al. Abnormal Resting-State Brain Activities in Patients with First-Episode Obsessive-Compulsive Disorder. *Neuropsychiatric disease and treatment* (2017) 13:507-13. Epub 2017/03/01. doi: 10.2147/ndt.S117510.

8. Qiu L, Fu X, Wang S, Tang Q, Chen X, Cheng L, et al. Abnormal Regional Spontaneous Neuronal Activity Associated with Symptom Severity in Treatment-Naive Patients with Obsessive-Compulsive Disorder Revealed by Resting-State Functional Mri. *Neuroscience letters* (2017) 640:99-104. Epub 2017/01/21. doi: 10.1016/j.neulet.2017.01.024.

9. Giménez M, Guinea-Izquierdo A, Villalta-Gil V, Martínez-Zalacaín I, Segalàs C, Subirà M, et al. Brain Alterations in Low-Frequency Fluctuations across Multiple Bands in Obsessive Compulsive Disorder. *Brain imaging and behavior* (2017) 11(6):1690-706. Epub 2016/10/25. doi: 10.1007/s11682-016-9601-y.

10. Zhao HZ, Wang CH, Gao ZZ, Ma JD, Huang P, Li HF, et al. Effectiveness of Cognitive-Coping Therapy and Alteration of Resting-State Brain Function in Obsessive-Compulsive Disorder. *Journal of affective disorders* (2017) 208:184-90. Epub 2016/10/30. doi: 10.1016/j.jad.2016.10.015.

11. Fan J, Zhong M, Gan J, Liu W, Niu C, Liao H, et al. Spontaneous Neural Activity in the Right Superior Temporal Gyrus and Left Middle Temporal Gyrus Is Associated with Insight Level in Obsessive-Compulsive Disorder. *Journal of affective disorders* (2017) 207:203-11. Epub 2016/10/11. doi: 10.1016/j.jad.2016.08.027.

12. Li K, Zhang H, Yang Y, Zhu J, Wang B, Shi Y, et al. Abnormal Functional Network of the Thalamic Subregions in Adult Patients with Obsessive-Compulsive Disorder. *Behavioural brain research* (2019) 371:111982. Epub 2019/05/30. doi: 10.1016/j.bbr.2019.111982.

13. Xia J, Fan J, Du H, Liu W, Li S, Zhu J, et al. Abnormal Spontaneous Neural Activity in the Medial Prefrontal Cortex and Right Superior Temporal Gyrus Correlates with Anhedonia Severity in Obsessive-Compulsive Disorder. *Journal of affective disorders* (2019) 259:47-55. Epub 2019/08/23. doi: 10.1016/j.jad.2019.08.019.

14. Yang X, Hu X, Tang W, Li B, Yang Y, Gong Q, et al. Intrinsic Brain Abnormalities in Drug-Naive Patients with Obsessive-Compulsive Disorder: A Resting-State Functional Mri Study. *Journal of affective disorders* (2019) 245:861-8. Epub 2019/02/01. doi: 10.1016/j.jad.2018.11.080.

15. Hu X, Zhang L, Bu X, Li H, Li B, Tang W, et al. Localized Connectivity in Obsessive-Compulsive Disorder: An Investigation Combining Univariate and Multivariate Pattern Analyses. *Frontiers in behavioral neuroscience* (2019) 13:122. Epub 2019/06/30. doi: 10.3389/fnbeh.2019.00122.

16. Gao J, Zhou Y, Yang X, Luo J, Meng F, Zheng D, et al. Abnormalities within and Beyond the Cortico-Striato-Thalamo-Cortical Circuitry in Medication-Free Patients with Ocd Revealed by the Fractional Amplitude of Low-Frequency Fluctuations and Resting-State Functional Connectivity. *Neuroscience letters* (2019) 712:134449. Epub 2019/08/31. doi: 10.1016/j.neulet.2019.134449.

17. Yang X, Luo J, Zhong Z, Yang X, Yao S, Wang P, et al. Abnormal Regional Homogeneity in Patients with Obsessive-Compulsive Disorder and Their Unaffected Siblings: A Resting-State Fmri Study. *Frontiers in psychiatry* (2019) 10:452. Epub 2019/07/19. doi: 10.3389/fpsyt.2019.00452.

18. Xia J, Fan J, Liu W, Du H, Zhu J, Yi J, et al. Functional Connectivity within the Salience Network Differentiates Autogenous- from Reactive-Type Obsessive-Compulsive Disorder. *Progress in neuro-psychopharmacology & biological psychiatry* (2020) 98:109813. Epub 2019/12/01. doi: 10.1016/j.pnpbp.2019.109813.

19. Gürsel DA, Reinholz L, Bremer B, Schmitz-Koep B, Franzmeier N, Avram M, et al. Frontoparietal and Salience Network Alterations in Obsessive–Compulsive Disorder: Insights from Independent Component and Sliding Time Window Analyses. *Journal of psychiatry & neuroscience : JPN* (2020) 45(3):214-21. Epub 2020/03/14. doi: 10.1503/jpn.190038.

20. Long J, Luo L, Guo Y, You W, Li Q, Li B, et al. Altered Spontaneous Activity and Effective Connectivity of the Anterior Cingulate Cortex in Obsessive–Compulsive Disorder. *Journal of Comparative Neurology* (2021) 529(2):296-310. doi: 10.1002/cne.24948.

21. Yu XM, Qiu LL, Huang HX, Zuo X, Zhou ZH, Wang S, et al. Comparison of Resting-State Spontaneous Brain Activity between Treatment-Naive Schizophrenia and Obsessive-Compulsive Disorder. *BMC psychiatry* (2021) 21(1):544. Epub 2021/11/05. doi: 10.1186/s12888-021-03554-y.

22. Liu J, Bu X, Hu X, Li H, Cao L, Gao Y, et al. Temporal Variability of Regional Intrinsic Neural Activity In drug-Naïve Patients with Obsessive–Compulsive Disorder. *Human brain mapping* (2021) 42(12):3792-803. doi: 10.1002/hbm.25465.

23. Zhang Y, Liao J, Li Q, Zhang X, Liu L, Yan J, et al. Altered Resting-State Brain Activity in Schizophrenia and Obsessive-Compulsive Disorder Compared with Non-Psychiatric Controls: Commonalities and Distinctions across Disorders. *Frontiers in psychiatry* (2021) 12:681701. Epub 2021/06/08. doi: 10.3389/fpsyt.2021.681701.

24. Yan H, Shan X, Li H, Liu F, Guo W. Abnormal Spontaneous Neural Activity as a Potential Predictor of Early Treatment Response in Patients with Obsessive–Compulsive Disorder. *Journal of affective disorders* (2022) 309:27-36. doi: 10.1016/j.jad.2022.04.125.

25. Han S, Xu Y, Guo HR, Fang K, Wei Y, Liu L, et al. Two Distinct Subtypes of Obsessive Compulsive Disorder Revealed by a Framework Integrating Multimodal Neuroimaging Information. *Human brain mapping* (2022) 43(14):4254-65. Epub 2022/06/22. doi: 10.1002/hbm.25951.

26. Yan H, Shan X, Li H, Liu F, Guo W. Abnormal Spontaneous Neural Activity in Hippocampal–Cortical System of Patients with Obsessive–Compulsive Disorder and Its Potential for Diagnosis and Prediction of Early Treatment Response. *Frontiers in cellular neuroscience* (2022) 16 C7 - 906534. doi: 10.3389/fncel.2022.906534.

27. Yu J, Xie M, Song S, Zhou P, Yuan F, Ouyang M, et al. Functional Connectivity within the Frontal–Striatal Network Differentiates Checkers from Washers of Obsessive-Compulsive Disorder. *Brain sciences* (2022) 12(8 C7 - 998). doi: 10.3390/brainsci12080998.

28. Tomiyama H, Murayama K, Nemoto K, Hasuzawa S, Mizobe T, Kato K, et al. Alterations of Default Mode and Cingulo-Opercular Salience Network and Frontostriatal Circuit: A Candidate Endophenotype of Obsessive-Compulsive Disorder. *Progress in neuro-psychopharmacology & biological psychiatry* (2022) 116. doi: 10.1016/j.pnpbp.2022.110516.

29. Ma Y, Zhao Q, Xu T, Wang P, Gu Q, Wang Z. Resting State Functional Brain Imaging in Obsessive-Compulsive Disorder across Genders. *The world journal of biological psychiatry : the official journal of the World Federation of Societies of Biological Psychiatry* (2022) 23(3):191-200. Epub 2021/09/04. doi: 10.1080/15622975.2021.1938669.

30. Yuan X, Zhu Y, Xiao L, Chuan Liu Z, Zou J, Hu Z, et al. Regional Homogeneity in Patients with Obsessive–Compulsive Disorder and Depression: A Resting State Functional Magnetic Resonance Imaging Study. *Neuroscience letters* (2023) 817 C7 - 137528. doi: 10.1016/j.neulet.2023.137528.

31. Wu X, Yang Q, Xu C, Huo H, Seger CA, Peng Z, et al. Connectome-Based Predictive Modeling of Compulsion in Obsessive-Compulsive Disorder. *Cerebral cortex (New York, NY : 1991)* (2023) 33(4):1412-25. Epub 2022/04/21. doi: 10.1093/cercor/bhac145.
