# Supplementary material for: Convergent functional change of frontoparietal network in obsessive-compulsive disorder: a voxel-based meta-analysis
Source: Front Psychiatry. 2024 Jul 8;15:1401623. doi: 10.3389/fpsyt.2024.1401623 (PMC11260709; doi:10.3389/fpsyt.2024.1401623)
Supplement: Supplementary file 2 [file Table_2.docx]

Jackknife sensitivity analysis of ALFF/fALFF

| Analysis | Hyperconnectivity region | | | | Hypoconnectivity region | |
| --- | --- | --- | --- | --- | --- | --- |
| Discarded dataset | Left MFG | Left IFG | Left STG | Left insula | Right IPL | Right precuneus |
| Hou et al. 2012 | YES | YES | YES | YES | YES | YES |
| Cheng et al. 2013 | YES | YES | YES | YES | NO | NO |
| Qiu et al. 2017 | YES | YES | YES | YES | YES | YES |
| Giménez et al. 2017 | NO | NO | NO | NO | NO | NO |
| Zhao et al. 2017 | YES | YES | YES | YES | YES | YES |
| Fan et al. 2017 | YES | YES | YES | YES | YES | YES |
| Xia et al. 2019 | NO | NO | NO | NO | YES | YES |
| Gao et al. 2019 | YES | YES | YES | YES | YES | YES |
| Yang et al. 2019 | YES | YES | YES | YES | YES | YES |
| Li et al. 2019 | YES | YES | YES | YES | YES | YES |
| Yu et al. 2021 | YES | YES | YES | YES | NO | NO |
| Liu et al. 2021 | YES | NO | NO | NO | NO | NO |
| Yan et al. 2022a | NO | YES | YES | YES | YES | YES |
| Han et al. 2022 | YES | YES | YES | YES | YES | YES |
| Ma et al. 2022 | YES | YES | YES | YES | YES | YES |

**Abbreviations:** MFG, medial frontal gurus; IFG, inferior frontal gyrus; STG, superior temporal gyrus; IPL, inferior parietal lobule;

Jackknife sensitivity analysis of ReHo

| Analysis | Hyperconnectivity region | | | Hypoconnectivity region | | | |
| --- | --- | --- | --- | --- | --- | --- | --- |
| Discarded dataset | Left precuneus | Right precuneus | Left cuneus | Left caudate body | Left caudate head | Right caudate body | Right PCC |
| Yang et al. 2010 | YES | YES | YES | YES | YES | YES | YES |
| Ping et al. 2013 | YES | YES | YES | YES | YES | YES | YES |
| Yang et al. 2015 | YES | NO | YES | YES | YES | NO | YES |
| Niu et al. 2017 | YES | YES | YES | YES | YES | YES | YES |
| Yang et al. 2019 | YES | YES | YES | YES | YES | YES | YES |
| Hu et al. 2019 | YES | NO | NO | YES | YES | YES | YES |
| Xia et al. 2020 | YES | YES | YES | YES | YES | YES | YES |
| Yu et al. 2021 | YES | YES | YES | YES | YES | YES | YES |
| Yan et al. 2022b | YES | YES | YES | YES | YES | YES | YES |
| Yu et al. 2022 | YES | YES | YES | YES | YES | YES | YES |
| Yuan et al. 2023 | YES | YES | YES | YES | YES | NO | YES |

**Abbreviations:** MTG, middle temporal gyrus; PCC, posterior cingulate cortex.

Jackknife sensitivity analysis of FC

| Analysis | Hyperconnectivity region | | Hypoconnectivity region | | | |
| --- | --- | --- | --- | --- | --- | --- |
| Discarded dataset | Right CG | Right Thalamus | Left DLPFC | Left IFG | Right DLPFC | Right Precentral Gyrus |
| Cheng et al. 2013 | YES | YES | YES | YES | YES | YES |
| Chen et al. 2016 | YES | YES | YES | YES | YES | YES |
| Gao et al. 2019 | YES | YES | YES | YES | YES | YES |
| Yang et al. 2019 | YES | YES | YES | YES | YES | YES |
| Gürsel et al. 2020 | YES | YES | YES | YES | YES | YES |
| Zhang et al. 2021 | NO | NO | YES | YES | YES | YES |
| Long et al. 2021 | YES | YES | YES | YES | YES | YES |
| Tomiyama et al. 2022 | YES | NO | YES | YES | YES | YES |
| Ma et al. 2022 | YES | YES | YES | YES | NO | NO |
| Yu et al. 2022 | YES | YES | YES | YES | YES | YES |
| Wu et al. 2023 | YES | YES | YES | YES | NO | NO |

**Abbreviations:** Cg, cingulate gyrus; DLPFC, dorsolateral prefrontal cortex; IFG, inferior frontal gyrus.
